# A Critical Review of Alien Limb-Related Phenomena and Implications for Functional Magnetic Resonance Imaging Studies

**DOI:** 10.3389/fneur.2021.661130

**Published:** 2021-09-09

**Authors:** Martina Di Pietro, Mirella Russo, Fedele Dono, Claudia Carrarini, Astrid Thomas, Vincenzo Di Stefano, Roberta Telese, Laura Bonanni, Stefano L. Sensi, Marco Onofrj, Raffaella Franciotti

**Affiliations:** ^1^Department of Neuroscience, Imaging and Clinical Sciences, “G. D'Annunzio” University of Chieti-Pescara, Chieti, Italy; ^2^Center for Advanced Studies and Technology (CAST), “G. D'Annunzio” University, Chieti, Italy; ^3^Department of Biomedicine, Neuroscience and Advanced Diagnostic (BiND), University of Palermo, Palermo, Italy; ^4^IRCCS C. Mondino Foundation, Pavia, Italy; ^5^YDA Foundation, Institute of Immune Therapy and Advanced Biological Treatment, Pescara, Italy

**Keywords:** alien hand, alien limb, neurodegeneration, corticobasal syndrome, diagonistic dyspraxia

## Abstract

Consensus criteria on corticobasal degeneration (CBD) include alien limb (AL) phenomena. However, the gist of the behavioral features of AL is still “a matter of debate.” CBD-related AL has so far included the description of involuntary movements, frontal release phenomena (frontal AL), or asomatognosia (posterior or “real” AL). In this context, the most frequent symptoms are language and praxis deficits and cortical sensory misperception. However, asomatognosia requires, by definition, intact perception and cognition. Thus, to make a proper diagnosis of AL in the context of CBD, cognitive and language dysfunctions must be carefully verified and objectively assessed. We reviewed the current literature on AL in CBD and now propose that the generic use of the term AL should be avoided. This catchall AL term should instead be deconstructed. We propose that the term AL is appropriate to describe clinical features associated with specific brain lesions. More discrete sets of regionally bound clinical signs that depend on dysfunctions of specific brain areas need to be assessed and presented when posing the diagnosis. Thus, in our opinion, the AL term should be employed in association with precise descriptions of the accompanying involuntary movements, sensory misperceptions, agnosia-asomatognosia contents, and the presence of utilization behavior. The review also offers an overview of functional magnetic resonance imaging-based studies evaluating AL-related phenomena. In addition, we provide a complementary set of video clips depicting CBD-related involuntary movements that should not mistakenly be interpreted as signs of AL.

## Introduction

The corticobasal syndrome (CBS) is a complex and progressive clinical picture featured by progressive asymmetric rigidity with apraxia, associated with the variable presence of cortical sensory symptoms, cortical myoclonus, alien limb (AL) phenomena, aphasia, cognitive disorders, dysarthria, bradykinesia, and tremor. CBS was initially described as corticobasal degeneration [CBD formerly known “cortical basal ganglionic degeneration” (1)], a condition characterized by progressive atrophy of frontotemporal regions and typical tau-containing inclusions known as “coiled bodies” and “astrocytic plaques” (2).

A syndromic definition was eventually found to be more appropriate, as distinct CBD-related pathology is only present in <40% of subjects exhibiting CBS signs. In context, several CBS cases exhibit pathology indicative of Alzheimer's disease, frontotemporal lobar degeneration (FTLD), Creutzfeldt–Jakob disease, and progressive supranuclear palsy (1, 3, 4).

The reviewed criteria for the diagnosis of CBS and CBD report that AL phenomena are described in 30% of CBD patients (5). These phenomena were also included in prior criteria (6), but the core features of AL remain a “matter of debate.” Despite this inconsistency, the recent criteria have identified AL as “complex unintentional limb movements interfering with normal tasks and the sensation that a limb is foreign.” The new criteria also stressed that AL is “more than simple levitation.” Interestingly, a previous report (7), authored by many researchers who contributed to the recent criteria (1), did not consider, as part of the AL phenomena, the sensation of foreignness but just the unintentional movement.

In the present article, we have reviewed the AL literature related to patients exhibiting CBS signs. We argue that the generic use of the term is conceptually inappropriate. We propose that the catchall term of AL should be deconstructed in more detail. Regionally bound sets of clinical signs should be appropriately identified and correctly grouped more coherently. In that regard, neurophysiology or neuroimaging studies can significantly help dissect the brain regions involved in producing distinct phenomena that cannot be grouped under the simplistic term of AL. The paper also includes a summary of functional magnetic resonance imaging (fMRI) studies investigating involuntary movements. We also include clinical video clips of involuntary movements present in CBS that should not be interpreted as AL.

## Alien Limb Phenomenology

AL had been described, under different terms, as a stable phenomenon in patients with destructive–non-progressive lesions of frontal or parietal areas or the corpus callosum.

In 1967, Rebeiz et al. produced the first report on CBD. Interestingly, in the original report, the presence of AL was not mentioned (8, 9). In the late '80s, descriptions of involuntary movements, with levitation, finger writhing, grasping, and groping, were again indicated as CBD-related clinical features (10–12).

In 1992, Doody and Jankovich (13) proposed a definition of AL in a study on seven patients, four of whom had (possibly) CBD, one CBD plus stroke-related infarction, and two stroke-related infarctions (posterior-temporal and bilateral fronto-parietal). The study proposed that AL should be “reserved for cases in which the hand feels foreign together with observable involuntary movements.” The core concept that “the hand feels foreign” or “has a will of its own” was substantiated by the described patient's perception that “their limb either did not obey them or that it did not belong to them.” This AL definition resurfaced in several case reports, case series, and CBS reviews (14–17).

**Figure 1:**
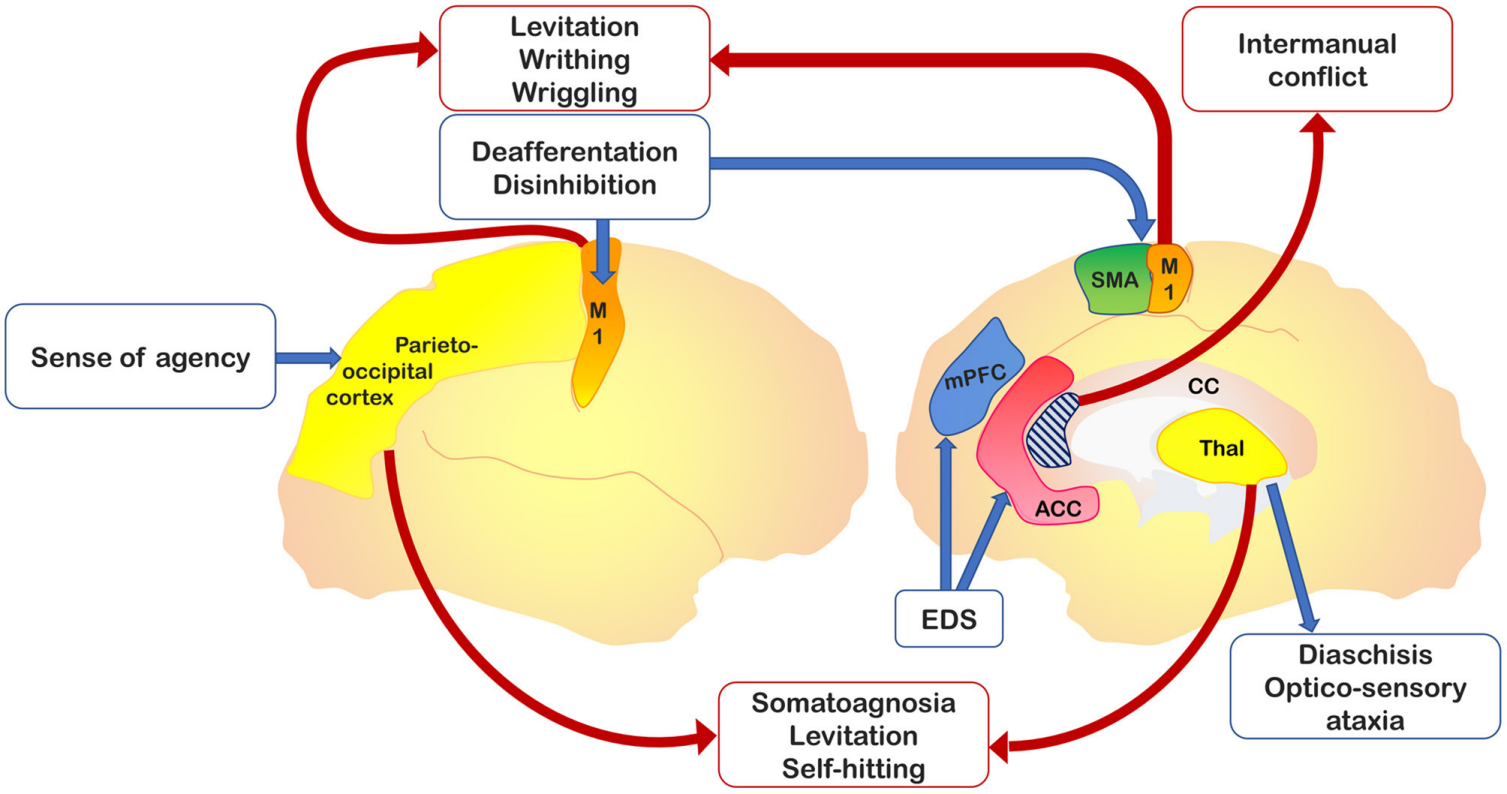
Relevant brain areas associated with alien hand–alien limb (AH-AL) variants and motor control. In yellow, the parieto-occipital and thalamic regions, which are damaged in posterior variant; in dark blue, the medial prefrontal cortex, impaired in the frontal variant; in light blue, the corpus callosum, whose anterior region is involved into the callosal variant; in orange, the primary motor cortex, whose isolated activation has been documented in involuntary movements; in green, the supplementary motor area (SMA) involved in internal drives of motor planning; and in red, the anterior cingulate cortex, implicated in action inhibition.

It should be stressed that AL as a phenomenological gist has been described as associated with vascular or neoplastic lesions of the supplementary motor area (SMA), anterior cingulate, corpus callosum, anterior prefrontal cortex, parietal cortex, and thalamus (18). AL-related symptoms have been categorized initially into two, then into three, distinct variants (19): 1) a posterior, “sensory” type, also called “real AL,” which consists of the patient's subjective feeling that his/her hand does not belong to himself/herself; 2) an anterior, “frontal” type consists of goal-directed movements that the patient does not perceive as initiated or controlled by his own will; and 3) a callosal type, or “diagonistic dyspraxia,” that consists of actions that interfere with the ones made by the contralateral hand. The latter variant sometimes appeared misspelled in the literature as “diagnostic dyspraxia” (13, 16), rather than the original “diagonistic” (12). However, this categorization appears to be not exhaustive, as parietal damage was found in patients presenting with limb levitation (20–23), and thalamic acute lesions may present with features of the posterior variant of AL (i.e., levitation movement of the affected limb and sense of extraneousness), despite the preservation of parietal areas (24–26). Figure 1 shows relevant brain areas involved in AL.

The overall picture is, therefore, confusing. For instance, some authors have indicated that the AL term is improperly used when describing proper CBS-related clinical features (27, 28). They correctly argue that the term has been employed to illustrate phenomena like purposeless movements, spontaneous levitation of the arm, or tentacular movements of the fingers with little in common with genuine AL.

The use of the term “anarchic hand” to identify “frontal” variant and the term “alien hand” for “posterior” variant (28) has also been proposed. A recent review paper (29) has provided an algorithm to disentangle AL types. The study has used the main accompanying signs for each AL type to subdivide the condition in 1) levitation-clumsiness (apraxia), foreignness, and neglect for the posterior variant; 2) groping, grasping, and other frontal signs for the frontal variant; and 3) limb conflicting with opposite limb and callosal disconnection signs for the callosal variant. This last review included CBS among the causes of AL but, in agreement with studies on lesional AL (18, 27, 28), stated that the sense of foreignness is only appropriate in posterior variant cases, thereby indicating that the patient's feeling of foreignness-extraneousness cannot semiotically be put together with the unwillingness of movement.

## Lesional vs. Neurodegenerative Alien Limb

Posterior variant's key symptom of AL is the sense of foreignness, extraneousness, and alienness of a limb. Thus, the symptom is comparable with asomatognosia and, like all agnosias, should be considered dependent on parietal or temporal lobe dysfunctions. Operational definitions state that “alertness, intelligence and normal command of language are prerequisites for the diagnosis of agnosia” together with “normal perception,” as “it is difficult to determine whether a patient perceives stimuli as well as the examiner does” (30). However, in CBS, parietal signs, like neglect, body schema dysfunctions, sensory extinction, agraphesthesia, and astereognosis, are part of the cortical sensory syndrome; and cognitive alterations are expected. Therefore, it is the underlying concept of agnosia that makes it untenable, applying the term “posterior AL” to CBS. Furthermore, as stated by Goldberg and colleagues, “it is now clear that cognitive and language disturbances are prominent in CBS” (31). Thus, the terms “posterior AL” and “real AL” are appropriate for isolated destructive lesions but questionable when employed in the context of CBS and other neurodegenerative conditions. The identification of AL requires that concomitant and undergoing subtle language and cognitive defects are excluded. Therefore, the inclusion of AL as a symptom occurring in CBS and other neurodegenerative diseases is misleading and inappropriate. It should be replaced by an appropriate description of the observed involuntary movements.

The progressive nature of diseases at the origin of CBS also casts doubts on the essence of the anterior–frontal AL definition in this clinical picture. Frontal lobe disorders (destructive lesions and frontotemporal dementia (FTD) spectrum disorders) are associated with utilization or imitation behavior (UB-IB)—also termed stimulus-bound behavior or environmental dependency syndrome (EDS)—that consists of semiautomatic behaviors that are produced by patients in the presence of objects or in response to stimuli provided by the examiner (32, 33). UB-IB depends on disinhibition and reduced volition that lead to the production of actions. Frontal lobe dysfunctions encompass a variety of clinical signs like 1) UB-IB as manifestations of higher complexity or more straightforward actions like touching the examiner's hand and following his movements, or 2) fondling blankets or clothes as intermediate-level manifestations or planning, or 3) grasping and groping as lower-order manifestations (34). However, patients do not experience these behaviors as “alien,” probably due to the frontal system's damage (35). The latest reviews (29, 31) on CBS and AL explicitly indicate the occurrence of frontal lobe symptoms in CBS, as expected, at least in cases with underlying FTLD-related pathology. Therefore, some of CBS's motor activities should be ascribed to UB-IB rather than the frontal AL. Moreover, the presence of “cognitive and language disturbances” may hinder the understanding of foreignness or “will of its own.”

As discussed in the next section, variable mixtures of UB-IB, involuntary movements, levitation, and apraxia have been described in CBS. Still, ratings of UB-IB, inhibition, and even the effect of entrainment maneuvers-distraction were rarely or never detailed.

## Theoretical Mainframes for Alien Limb

It appears evident that the different signs described as AL in the context of CBS cannot be considered as such. A throughout analysis of the undergoing AL mechanisms in subjects exhibiting regional degeneration of the parietal, frontal, and callosal areas indicates alternative and more viable options (Figure 1). AL should be deconstructed in a set of regionally bound signs driven by the dysfunction of specific brain areas. In the context of parietal AL, disconnections from the medial paralimbic system need to be ascertained, and callosal disconnections should be investigated in the presence of callosal signs (intermanual conflict). Grasping or reaching, UB-IB, and mirror movements depend on dysfunctions of structures involved in planning and storage of motor subroutines internally driven (i.e., involving the SMA) or responsive to movements (i.e., involving the premotor medial system or premotor cortex) (28). Therefore, these areas should be carefully investigated when dealing with AL patients to generate a regionally localized description of distinct clinical features. A theoretical interpretation of the frontal AL can consider the interaction between the medial and lateral premotor systems, which are the functional drivers of voluntary actions. Damage of the medial premotor system can disinhibit the lateral premotor system and generate “stereotypical” actions (36). An alternative theoretical construct underlines the role of fronto-parietal networks in selecting, among all the possible options, voluntary actions to be performed in response to an external stimulus (37, 38). This selection process is influenced by a decision-making system that encompasses the prefrontal and striatal circuits. In frontal AL, damage of frontal regions compromises the brain's ability to select or inhibit a behaviorally relevant action, thereby leading to the exaggerated representation of potential actions activated by external sensory stimuli (39).

One of the newest theoretical models employs the Bayesian framework for motor symptoms. The model considers a hierarchy of three levels: 1) an upper level of abstract and self-referential beliefs associated with the activity of the rostral and lateral prefrontal cortices; 2) an intermediate level involving the interaction between the medial and lateral premotor systems to optimize the relative precision of prior beliefs and sensory inputs; and 3) a lower level that resides in the spinal motor neurons where proprioceptive predictions from the upper motor neurons are compared with proprioceptive inputs, thereby generating the initiating of the movement. Thus, according to this Bayesian approach, AL should be considered as resulting from a discrepancy between an intact upper level and an imprecise intermediate level. The mismatch is thought to produce a failure to integrate top-down predictions with bottom-up prediction errors (40).

## Corticobasal Syndrome-Related Alien Limb: A Critical Review

Table 1 reports a synopsis of findings on AL in CBS described in different studies (7, 10, 13–17, 39, 41, 42, 44, 45). Only few reports are structured as case series or cohorts (7, 13, 14, 17, 42); many are single case studies (10, 15, 16, 39, 41). Two of the most recent studies (44, 45) explored presence and features of AL in a cohort of patients affected by CBS. Albrecht et al. (45) indicated “alien limb” or “posterior variant” conditions in patients who experienced the feeling that the limb was unfamiliar or did not belong to them. The term “anarchic limb” or “frontal variant” for involuntary but goal-directed movements has been instead used (28) to emphasize the specific and different origins of these disturbances (i.e., the postcentral gyrus and somatosensory cortex, or the SMA and medial prefrontal cortex, respectively) related to the abnormalities in awareness and control of actions (46).

**Table 1:** Alien hand in corticobasal syndrome: previous case reports and case series.

**Author**	**Wenning et al. (7)**	**Rebeiz et al. (8)**	**Gibb et al. (10)**	**Riley et al. (11)**	**Doody and Jankovic (13)**	**Rinne et al. (14)**	**Fitzgerald et al. (15)**	**Schaefer et al. (16)**	**Graff-Radford et al. (17)**	**Schaefer et al. (41)**	**McBride et al. (39)**	**Vanek et al. (42)**	**Onofrj et al. (43)**	**Lewis-Smith et al. (44)**	**Albrecht et al. (45)**
N°Cases	4[1]	2	1[4]	10	5	14[2]	1	1	108[3]^**^	1	1	34	4	25/30	8/25
Limb	Not known	Center	Center	8 center 2 right	3 right 2 center	Not known	Right	Center	65% center	Right	Right	Not known	3 center 1 right	Not known	Not known
Term used	Alien hand		Alien hand	Alien limb	Alien hand	Classical alien limb	Alien limb sign	Alien limb syndrome	Alien limb syndrome	Alien hand syndrome	Mild alien hand behavior	Alien limb phenomena	Levitation and tentacular movements	Alien limb phenomena	Alien limb/ anarchic limb
Involontary movements	4/4	Yes		Yes	Yes			Yes		Yes	Yes	Yes		11/30	4/30
Levitation/ posturing		Yes	Yes	Yes (2/10)	3/5		Yes				No		Yes	14/30	
Tentacular movements				1/10									Yes		
Limb wandering			Yes			Yes									
Intermanual conflict						Yes	No	Yes			No			5/30	
Frontal signs (pathological grasp, utilization behavior)		Yes		6/10	4/5 grasping 1/5 utilization behavior	Yes	Yes	Yes	7%^*^	Yes	Yes				
Neglect									16%						
Mirror movements		Yes			1/5		No	No	41%	Yes	No			10/10	
Myoclonus				9/10	4/5			Yes	32%	Yes			No		
Dystonia	3/4	Yes		7/10					49%	Yes	No	100%	Yes		
Apraxia	3/4	Yes		8/10		Yes	Yes		96%	Yes	No		Yes	29/30	
Rigidity		Yes		10/10	5/5	Yes		Yes	94%	Yes	Yes		Yes		
Cortical sensory symptoms	1/4	Yes		3/10				No	44%	Yes	No		Yes	Astereoagnosia (15/30)	5/30
Choreiform movements			Yes												

*In brackets the definition, when described, given by the author: [1] The patients found their arm wandering uncontrollably, sometimes crossing the midline and interfering with the movement of the contralateral limb, and sometimes grasping onto objects. They described their limb as 'having a mind of its own' or 'it just does not do what I want it to do'. Some of these patients described the affected limb as being foreign to them, 'it does not belong to me', but others felt the limb to be part of their body image but beyond their control. [2] Complex unintentional movements interfering with normal motor tasks. [3] Inclusion criteria: Doody-Jankovic diagnostic criteria: the patient experienced that an extremity “is foreign or 'has a will of its own,' together with observable involuntary motor activity. [4] Difficulty in using her center hand, which levitates, wanders, and floats uncontrollably. She often falls and has superimposed choreiform movements of the limbs*.

* *Frontal/callosal alien hand in 7%*;

** *predominance of the so-called posterior phenotype*.

*YES, indicates that the phenomenon is reported in the paper but no percentage is described in detail. NO, indicates that the phenomenon is expressly described as absent in all the patient*.

A better clarification of the AL term's symptoms has been provided by the study questionnaires filled out by patients or caregivers (44). The most common descriptions were “tend to hold the offending hand with the better hand” (50% of CBD patients), followed by “unwilled arm levitation” (47%) and/or “sensation of foreignness of their limb” (50%). The authors found no association between AL-related phenomena and limb apraxia severity, thereby suggesting that AL associated with neurodegenerative diseases is not driven by apraxia.

Moreover, three studies (13, 17, 28) investigated among AL cases how many had CBS. Apart from single case descriptions (10, 15, 16, 39, 41) in most studies, it is difficult to determine the coexistence of symptoms, i.e., whether patients with apraxia also show levitation and grasping, or patients with posterior AL also exhibit cortical sensory symptoms. For instance, a study using the AL definition proposed by Doody and Jankovic (13) reported dystonia, rigidity, and mirror movements in 41% and intermanual conflict in 7% of CBS, thereby leaving one with the idea that the remaining patients exhibited posterior AL. However, 40% of CBS patients showed neglect, and many exhibited language disorders and cortical sensory syndromes (17).

Levitation of the arm, tentacular finger movements, athetosis, finger writhing, wriggling of fingers and wrist, choreic and ballic movements have been described in several studies (10, 14, 16, 41), either framed into AL signs or described as separate entities. Still, no information was provided on the coexistence of these symptoms and other AL features. According to a review (3), levitation and tentacular finger movements occur only for “few months to few years,” disappearing when “severe dystonia and rigidity supersede,” but no information was offered about progression of choreic–ballic movements or somatoagnosia. Mirror movements have been described in recent papers (1, 31). Two single case studies (15, 16) described “triggered” AL, which consisted of levitation in response to being touched by the examiner or sticking a finger to the examiner's finger and following the movements of the examiner's hand.

## fMRI Findings on Alien Limb Phenomena

The few fMRI studies that have investigated AL have produced contradictory results. The most recent review on AL (29) took into consideration four imaging studies on “alien hand.” The study pool was a rather diverse mix. One study investigated a patient exhibiting movements induced by touching (41), the second one evaluated a patient with isolated finger writhing (47), and the third one evaluated four patients showing levitation and finger (and wrist) writhing (43). The final one investigated intentional binding in 10 patients with CBS (not all of whom had AL) and assessed changes in functional connectivity at rest and not upon movement (48). Only two fMRI studies (43, 47) specifically addressed levitation or tentacular athetoid-like movements. In one (47), the patient was classified as affected by AL secondary to parietal brain infarct. Still, the movement evaluated with fMRI consisted of finger writhing and wriggling of the dystonic arm. These involuntary movements were associated with isolated primary motor cortex (M1) activation and ascribed to disconnection from the parietal areas. A second study (43) was focused on fMRI correlates of levitation and tentacular movements produced by four patients (all with concomitant arm dystonia) and confirmed the presence of M1 activation. It should be underlined that the study also revealed that the activation of the complex motor network implicated in movement planning and execution upon involuntary movements involving the arm is also affected by involuntary movements (and dystonia).

Of note, the M1 activation was statistically higher in the affected side compared with the unaffected one. This finding could be explained as a failure of inhibitory control on the M1 area secondary to the asymmetric atrophy, which is a common feature of CBS patients (43). Therefore, in patients exhibiting levitation and tentacular movements with dystonia, the motor network is activated upon performing voluntary movements, despite the underlined motor disorders. The absence of the SMA activation upon involuntary movements confirmed the role of this region in the preparation and execution of willed movements (49).

In a CBS case described by Schaefer et al. (16), AL movements characterized by involuntary gestures or grasping were associated with the activation of the same areas that are typically involved upon the execution of voluntary movements (i.e., M1, premotor cortex, and precuneus). These movements also involved the activation of the inferior frontal gyrus. Therefore, the authors suggested that the inferior frontal gyrus is engaged in inhibitory control over involuntary motor responses.

The most recent imaging study on AL in CBS (50) produces a single-subject “atrophy network map” by using an atrophy map as a seed to investigate functional connectivity changes. The comparison between 16 CBS patients with and 25 CBS patients without AL identified a symptom-specific atrophy network map for AL in CBS. This network included the precuneus and the right temporal–parietal junction. The former was previously found in a study (51) using the lesion network induced by AL as seed for the functional connectivity analysis, while the latter was associated with agency in healthy subjects (52).

fMRI studies on healthy volunteers revealed the activation of multiple areas engaged to execute motor acts. These brain areas are summarized in Table 2.

**Table 2:** Activated areas during voluntary movements revealed by fMRI studies performed on healthy volunteers.

**Areas**	**No. of studies**	**fMRI studies**
M1	9	Allison et al. (53); Ball et al. (54); Baraldi et al. (55); Cunnington et al. (56); Cunnington et al. (57); Gerardin et al. (58); Hanakawa et al. (59); Joliot et al. (60); Sedov et al. (61)
SMA	6	Ball et al. (54); Cunnington et al. (56); Cunnington et al. (57); Joliot et al. (60); Nguyen et al. (49); Sedov et al. (61)
Pre-SMA	1	Cunnington et al. (62)
SI	5	Allison et al. (53); Cunnington et al. (57); Hanakawa et al. (59); Joliot et al. (60); Sedov et al. (61)
Premotor cortex	3	Baraldi et al. (55); Gerardin et al. (58); Sedov et al. (61)
Middle frontal gyrus	1	Joliot et al. (60)
Cingulate motor area	4	Ball et al. (54); Cunnington et al. (56); Cunnington et al. (57); Nguyen et al. (49)
Superior parietal cortex	2	Baraldi et al. (55); Cunnington et al. (56)
Inferior parietal lobe	2	Ball et al. (54); Joliot et al. (60)
Supramarginal gyrus	1	Joliot et al. (60)
Insula	3	Cunnington et al. (56); Joliot et al. (60); Sedov et al. (61)
Operculum	3	Hanakawa et al. (59); Joliot et al. (60); Sedov et al. (61)
Cerebellum	4	Gerardin et al. (58); Hanakawa et al. (59); Joliot et al. (60); Sedov et al. (61)
Striatopallidal complex	1	Sedov et al. (61)
Ventral thalamus	1	Sedov et al. (61)
Basal ganglia	2	Cunnington et al. (56); Gerardin et al. (58)

*SMA, supplementary motor area*.

## fMRI Findings During Other Involuntary Movements

A revision of the literature on involuntary movements indicates that fMRI studies mainly addressed essential tremor (ET), “tic” in Tourette's syndrome, and dystonia. Findings on ET suggested that tremor is generated by the same neural network that is activated upon voluntary movement (63, 64). Another fMRI study investigated ET patients and healthy controls maintaining a posture as well as controls simulating tremor (65). Tremor in ET patients was associated with decreased cerebellar, sensory-motor cortex, and basal ganglia activation compared with controls. A recent revision (66) offered an overview of functional imaging studies on Tourette's syndrome. The authors of the review acknowledged the incongruent findings, as one study showed increased (67, 68) and the other one decreased (69) activity of the pre-SMA and premotor cortex during putative involuntary movements. However, the studies converged in showing hyperactivation of the ipsilateral prefrontal cortex, anterior portion of the SMA, and contralateral cingulum (62, 67, 69).

An fMRI study (70) was conducted to characterize dystonia in which sustained muscle contractions lead to disabling repetitive movements or abnormal postures. The authors (70) indicated abnormal activity in premotor SMA and M1 during movement in both generalized and focal dystonias. Previous studies reported conflicting results and showed decreased (71) or increased activity (72, 73) in dystonic patients compared with controls.

Table 3 lists the brain regions activated upon involuntary movements as indicated by fMRI studies performed in patients with AL and in patients with other movement disorders (i.e., Huntington's disease and Parkinson's disease) where resting tremor and dyskinesia were mainly investigated by means of resting-state approaches (48, 86, 87) instead of using task-related fMRI.

**Table 3:** Activated areas during involuntary movements revealed by fMRI studies in patients with AL and other movement disorders.

**Areas**	**No. of studies**	**fMRI studies**
M1	11	Assal et al. (47); Berg et al. (64); Bohlhalter et al. (74); Boonstra et al. (75); Bucher et al. (63); Helmich et al. (76); Neuner et al. (77); Onofrj et al. (43); Schaefer et al. (16); van Rootselar et al. (78); Wu et al. (79)
S1	6	Berg et al. (64); Bohlhalter et al. (74); Boonstra et al. (75); Bucher et al. (63); Neuner et al. (77); van Rootselar et al. (78)
SMA	4	Bohlhalter et al. (74); Johnson and Gregory (80); Kloppel et al. (81); Neuner et al. (77)
Pre-SMA	3	Biswal et al. (68); Cerasa et al. (82); Roessner et al. (67)
Premotor cortex	4	Biswal et al. (68); Boonstra et al. (75); Roessner et al. (67); Schaefer et al. (16)
Frontal operculum	1	Boonstra et al. (75)
Thalamus	6	Berg et al. (64); Bohlhalter et al. (74); Bucher et al. (63); Helmich et al. (76); Neuner et al. (77); van Rootselar et al. (78)
Cerebellum	8	Berg et al. (64); Bohlhalter et al. (74); Boonstra et al. (75); Bucher et al. (63); Helmich et al. (76); Neuner et al. (77); Van Rootselar et al. (78); Wu et al. (79)
Insula	3	Bohlhalter et al. (74); Neuner et al. (77); van Rootselar et al. (78)
Inferior frontal gyrus	3	Cerasa et al. (83, 84); Schaefer et al. (16); van Rootselar et al. (78)
Superior temporal pole	1	van Rootselar et al. (78)
Superior parietal cortex	3	Kloppel et al. (81); Kloppel et al. (85); Wu et al. (79)
Anterior cingulate cortex	5	Biswal et al. (68); Bohlhalter et al. (74); Neuner et al. (77); Roessner et al. (67); Werner et al. (62)
Basal ganglia	6	Berg et al. (64); Bohlhalter et al. (74); Bucher et al. (63); Cerasa et al. (83, 84); Helmich et al. (76); Neuner et al. (77)
Red nucleus	2	Berg et al. (64); Bucher et al. (63)

*AL, alien limb; SMA, supplementary motor area*.

## Discussion

In this critical review, we posit that the generic term AL is often misleading and misplaced when applied to neurodegenerative diseases. AL-like phenomena must be dissected and replaced by appropriate descriptions of distinct features. We, therefore, suggest that the catchall term AL should be deconstructed and subdivided into three different entities: 1) levitation and writhing, likely related to involuntary activation and sensory deafferentation of M1; 2) absent “agency” (88) and/or hemisomatoagnosia, as a result of parietal lobe alterations; and 3) complex finalized movements with (3a) intermanual conflict or (3b) without intermanual conflict. Additional nosographic care should be applied. For instance, inter-manual conflict can be attributed to patients only if they exhibit the affected hand interfering with movements of the unaffected hand.

The attached videos show examples of involuntary movements, not considered as AL, observed in some patients of our case series with a diagnosis of CBS (Supplementary Materials).

The resting-state connectivity patterns are not strictly related to unwanted movements of the patients also when lesion network maps were used. According to the “lesion network overlap” concept (89), when lesions cause the same symptom in many patients and overlap in one brain region, the causal link between that region and the resulting symptom is strengthened. However, a distant lesion may induce morphological degenerations of other areas that are not involved in the area initially lesioned (the case of cerebellar olives and dentate nuclei in the most known clinical example), suggesting that functional connectivity alterations may induce degeneration due to disconnection (diaschisis) (90). Wallerian degeneration is another example (91). The often contradictory findings in unwanted action studies imply that these maladaptive responses (i.e., diaschisis and Wallerian degeneration) could influence the results.

The absence of fMRI evidence of which cortical or subcortical areas are active during any involuntary or non-volitional movements indicates that work still needs to be done on fMRI protocols. For instance, retrospectively analyzing acquired data related to involuntary movements (like in the back-averaging of electroencephalographic signals) can help dissect the issue. In addition, the quantitative analysis should be improved (92, 93) to exclude the possibility that differences between voluntary and involuntary movements may simply relate to differences, in intensity and/or regional extension, of activation within the same motor network.

Implementing fMRI methods to address involuntary movements could also help unravel the intrinsic mechanisms that lie beneath the production of consciousness (94).

Our review shows several inconsistencies. The most relevant relates to the fact that several studies on involuntary movements show the activation of the same brain areas activated by voluntary ones.

Future neuroimaging studies should implement three distinct categories:

1) lesional studies, separating levitation, somatoagnosia, and UB-IB;
2) studies aimed at dissecting the network specificity of consciousness correlates of the AL enactment; and
3) studies specifically addressing the frontal lobe role in conscious perception. These studies are needed as frontal lobe lesions disrupt the link between movements and consciousness. However, the disruption of the perception of self-agency rather than the perception of the body itself (like in asomatognosia) seems to be the major driving force. This fascinating hypothesis indeed requires further detailed investigation and experimental validation.

## Conclusions

The original AL definition (13) had the laudable effect of putting CBD on the radar screen. It also built the conceptual framework that has produced the systematization of the CBS category (4). However, it is now time to move forward and adequately identify clusters of independent signs and clinical features that have been wrongly put under the same inadequate AL banner. Thus, we propose that the term AL should be removed from the consensus-based list of symptoms employed for the CBS diagnosis. A list of detailed (involuntary or non-volitional) movements appearing in CBS, with accurate descriptions and quantitative approaches, should replace the term and settle the debate (5) on “what behaviors constitute alien limb phenomena” in CBS. Functional connectivity-based strategies can also help to dissect if any given “overlapping lesion” has a causal role in symptom production or simply plays a compensatory/adaptive role.

Levitation, tentacular movements, grasping, UB-IB, and mirror movements—so far described under the AL term—should be now defined in terms of patterns, co-occurrence, duration, frequency, and muscle involvement. The effect of entrainment or distractive maneuvers or interference should be analyzed. Once identified in their specificity, these movements should be the target of different investigational (neurophysiology and neuroimaging) and therapeutic approaches.

## Author Contributions

MDP revised the literature and drafted the manuscript. MR, FD, CC, AT, VDS, RT, LB, and SS revised the manuscript for intellectual content. MO and RF conceptualized the study, drafted, and revised the manuscript for intellectual content. All authors listed have made a substantial, direct and intellectual contribution to the work, and approved it for publication.

## Conflict of Interest

The authors declare that the research was conducted in the absence of any commercial or financial relationships that could be construed as a potential conflict of interest.

## Publisher's Note

All claims expressed in this article are solely those of the authors and do not necessarily represent those of their affiliated organizations, or those of the publisher, the editors and the reviewers. Any product that may be evaluated in this article, or claim that may be made by its manufacturer, is not guaranteed or endorsed by the publisher.
