# Supplementary material for: A Critical Review of Alien Limb-Related Phenomena and Implications for Functional Magnetic Resonance Imaging Studies
Source: Front Neurol. 2021 Sep 9;12:661130. doi: 10.3389/fneur.2021.661130 (PMC8458742; doi:10.3389/fneur.2021.661130)
Supplement: Supplementary file 6 [file Data_Sheet_1.docx]

**Supplementary materials**

**1.2 Supplementary Video**

All patients gave their informed consent to publish their video for scientific purposes.

**Supplementary Video 1.** Patient 1- CBS, final diagnosis: amyloid angiopathy. Athetoid (tentacular) movements of right hand and concomitant involuntary movements of the right lower limb/foot when sitting.

**Supplementary Video 2.** Patient 1- CBS, final diagnosis: amyloid angiopathy. Initial grasping when touching the patient’s finger, despite the examiner asks to restrain, then sticking to examiner finger during rotatory movements (phenomenon also defined as “Mitgehen”, ref 17 in text). Notice concomitant constraint of involuntary right foot movement by the left foot. Notice also that grasping is not suppressed when the patients arm is juxtaposed to body.

**Supplementary Video 3.** Patient 1- CBS, final diagnosis: amyloid angiopathy. Involuntary movements of the right arm and lower limb, notice unaffected arm and lower limb constraint (holding) of the affected limbs. While testing the utilization behavior, notice the involuntary movement of the lower limb, and the attempts to hold the limb.

**Supplementary Video 4.** Patient 2- CBS, final diagnosis: Progressive supranuclear palsy. Tentacular and levitation movements of the left hand with a choreic pattern. Notice restraintment maneuvers: the unaffected hand is holding the affected hand. This, and previous examples, are not examples of intermanual conflict, which could be identified only if the affected hand was holding the unaffected hand.

**Supplementary Video** 5 Patient 3- Corticobasal Degeneration. Right hand cortical myoclonus, with action myoclonus, right hand dystonia with flexion of wrist and fourth-fifth finger (Intrinsic Hand-Plus dystonia). Notice, during examination, grasping of the examiner's hand with relaxation only when the elbow is flexed. The hand of the patient appears sticking to the examiner's hand.
